# Relationship between retinal neurodysfunction and cognitive impairment in type 2 diabetes: results of the RECOGNISED cross-sectional study

**DOI:** 10.1007/s00125-025-06664-4

**Published:** 2026-01-29

**Authors:** Rafael Simó, Cristina Hernández, Simona Frontoni, Paolo Sbraccia, Reinier Schlingemann, Xavier Valldeperas, Stela Vujosevic, Inês Marques, José Cunha-Vaz, Jakob Grauslund, Frederik N. Pedersen, María-José Barahona, Natasa Popovic, Gianpaolo Zerbini, Andreea Ciudin, Santiago Perez-Hoyos, Lieza Exalto, Geert Jan Biessels, Noemi Lois

**Affiliations:** 1 Diabetes and Metabolism Research Unit, Vall d’Hebron Hospital Campus, Barcelona, Spain; 2 Centro de Investigación Biomédica en Red de Diabetes y Enfermedades Metabólicas Asociadas (CIBERDEM), Madrid, Spain; 3 Vall d’Hebron Research Institute and Autonomous University of Barcelona, Barcelona, Spain; 4 Department of Systems Medicine, Tor Vergata University, Rome, Italy; 5 Ocular Angiogenesis Group, Department of Ophthalmology, Amsterdam UMC location University of Amsterdam, Amsterdam, the Netherlands; 6 Department of Ophthalmology, Hospital Universitari Germans Trias i Pujol, Badalona, Spain; 7 Eye Clinic, IRCCS MultiMedica, Milan, Italy; 8 AIBILI - Association for Innovation and Biomedical Research on Light and Image, Coimbra, Portugal; 9 Department of Regional Health Research, University of Southern Denmark, Odense, Denmark; 10 Department of Endocrinology, Hospital Universitari MútuaTerrassa, University of Barcelona, Terrassa, Spain; 11 Department of Medical Physiology, Faculty of Medicine, University of Montenegro, Podgorica, Montenegro; 12 Diabetes Research Institute, IRCCS Istituto Scientifico San Raffaele, Milan, Italy; 13 Unit of Statistics and Bioinformatics, Vall d’Hebron Research Institute, Barcelona, Spain; 14 Department of Neurology and Neurosurgery, UMC Utrecht Brain Center, University Medical Center Utrecht, Utrecht University, Utrecht, the Netherlands; 15 Wellcome-Wolfson Institute for Experimental Medicine, School of Medicine, Dentistry and Biomedical Sciences, Queen’s University, Belfast, UK

**Keywords:** Cognitive impairment, Diabetic retinopathy, Electroretinography, Microperimetry, Mild cognitive impairment, Pupillary responses, Retinal neurodegeneration, Retinal neurodysfunction, Type 2 diabetes, Visuo-construction

## Abstract

**Aims/hypothesis:**

There are no robust, reliable and easy to administer tests to screen for mild cognitive impairment (MCI) in people living with diabetes. Since the retina is ontogenically brain-derived, we hypothesised that retinal biomarkers could be used, alone or in combination with other simple tests, to screen for MCI in people with diabetes.

**Methods:**

Baseline data from participants screened for RECOGNISED, a Horizon 2020-funded European project, were analysed. Main eligibility criteria for RECOGNISED included age ≥65 years, type 2 diabetes of over 5 years standing, no previous history of stroke or neurodegenerative disease, and no overt diabetic retinopathy or only mild-to-moderate non-proliferative diabetic retinopathy. Baseline characteristics of participants, including scores from the Montreal Cognitive Assessment test (MoCA) and Self-Administered Gerocognitive Examination, the Diabetes Specific Dementia Risk Score (DSDRS) and ophthalmological endpoints gathered from standardised seven field colour fundus photography, spectral domain optical coherence tomography, microperimetry and a hand-held portable electroretinography device (RETeval), were obtained and used in the work presented here as potential screening predictors for presence of MCI. MCI and normocognition (NC) were determined based on a full neuropsychological test battery and the Clinical Dementia Rating score. A stepwise selection of variables, based on Akaike’s information criterion, and logistic regression models for predicting MCI were undertaken. Area under the receiver-operating characteristic curve analyses were used to predict the probability of the presence of MCI as well as sensitivity and specificity cut-off points.

**Results:**

A total of 313 people living with diabetes (128 with NC and 185 with MCI) were included. People with diabetes with MCI were older (*p*=0.006) and had fewer years of education (*p*<0.001), lower retinal sensitivity (*p*=0.01) and less capacity of gaze fixation (*p*≤0.001) than those with NC. Statistically significant differences in pupillary area ratio (*p*=0.002) and photopic b-wave amplitude (*p*=0.03) were detected between people with diabetes with NC and with MCI. Multivariable logistic regression showed that the best model to identify people with diabetes with MCI was that combining retinal sensitivity, gaze fixation, photopic b-wave amplitude and pupillary size change following stimulation, years of education, DSDRS and MoCA score, with an AUC of 0.84 (sensitivity 79.9, specificity 79.0). The visuo-construction domain was the most affected in people with diabetes with MCI and its impairment was independently related to retinal sensitivity and gaze fixation.

**Conclusions/interpretation:**

The assessment of retinal neurodysfunction in combination with simple clinical variables appears useful to identify people with diabetes with MCI. This strategy could optimise current screening of MCI in people living with diabetes.

**Graphical Abstract:**

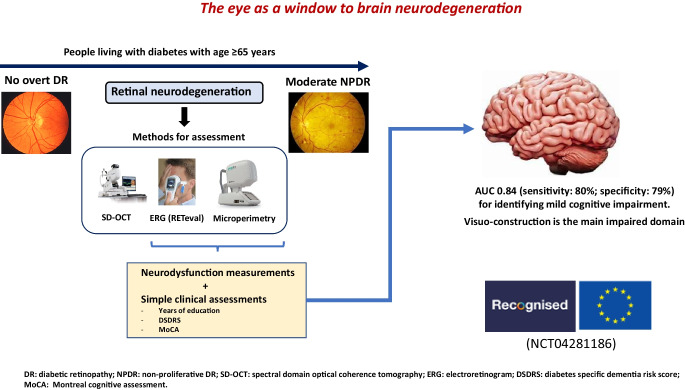

**Supplementary Information:**

The online version of this article (10.1007/s00125-025-06664-4) contains peer-reviewed but unedited supplementary material.


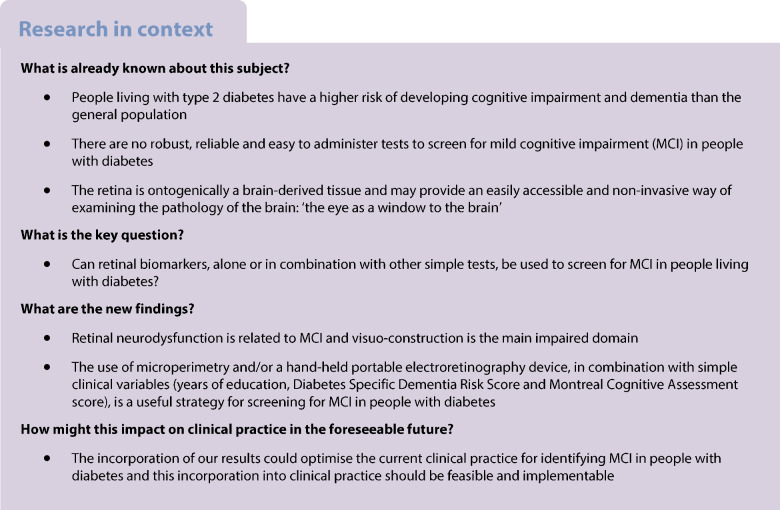


## Introduction

There is mounting evidence that type 2 diabetes is associated with cognitive impairment and dementia, and several epidemiological studies have shown that people living with type 2 diabetes have higher risk of developing Alzheimer’s disease in comparison with age-matched people without diabetes [1–4]. Notably, this increased risk remains significant after adjusting for cardiovascular risk factors [5, 6]. The impact of dementia on society as a whole is enormous [7], and the number of cases of dementia associated with type 2 diabetes is expected to increase due to the simultaneous rise of diabetes and age worldwide.

People living with type 2 diabetes have increased risk of developing mild cognitive impairment (MCI) [8–10], a critical transitional state between normal cognitive function and dementia. The annual conversion rate from MCI to dementia ranges between 10% and 15% in the general population [11, 12], but is significantly higher in people with type 2 diabetes [13, 14]. In this regard, we have shown that type 2 diabetes is a significant accelerator of dementia in people with MCI [14].

Cognitive impairment in people with type 2 diabetes affects self-management of the disease (e.g. medication, diet, blood glucose monitoring), thus favouring both hyperglycaemic peaks and episodes of hypoglycaemia. Consequently, the type 2 diabetes population with cognitive impairment has an increased frequency of hospital admissions and morbimortality [15, 16].

To date, there are no reported reliable screening examinations that could be used to identify people with diabetes with MCI, despite the fact that clinical guidelines recommend screening for cognitive impairment in type 2 diabetes [17, 18]. The diagnosis of MCI is based on multidisciplinary analyses which include time-consuming neuropsychological tests [19], making their incorporation into clinical practice for the type 2 diabetes population unfeasible. For this reason, several cost–benefit screening strategies based on less time-consuming tests such as the Mini Mental State Examination (MMSE) or the Montreal Cognitive Assessment (MoCA) have been recommended [20]. However, a recent meta-analysis highlighted the insufficient evidence supporting the MMSE as a screening tool to detect MCI [20]. Although MoCA seems more effective than MMSE for this purpose [21], this finding was based on a limited sample size (around 100 participants) and non-homogeneous cut-off. Furthermore, individuals having symptoms of depression were not included. This is a substantial bias, because a significant proportion of people living with type 2 diabetes older than 65 years have these symptoms, deleteriously affecting their quality of life (QoL) [22]. Self-administered neuropsychological questionnaires (i.e. Test Your Memory or the Self-Administered Gerocognitive Examination [SAGE]) have been proposed as case-finding strategies for cognitive impairment in people with diabetes in primary care [23], but they need further validation.

There is growing evidence that retinal neurodegeneration is an early pathophysiological condition in most individuals with diabetic retinopathy [24–26]. In addition, an association between retinal vessel abnormalities and cognitive impairment and dementia has been reported [27, 28]. Since the retina is embryologically a brain-derived tissue, it has been considered that it could be an accessible and non-invasive way to examine the pathology of the brain: ‘the eye as a window to the brain’ [16, 29, 30]. Therefore, it seems reasonable to hypothesise that retinal assessments related to either neurodegeneration or microvascular disease would be useful biomarkers to identify people living with type 2 diabetes at higher risk of developing cognitive impairment and dementia.

On this basis the European RECOGNISED consortium was created (‘Retinal and cognitive dysfunction in type 2 diabetes: unravelling the common pathways and identification of people at risk of dementia’) and funded by the European Commission (Horizon-2020; GA 847749), with the overarching aims of investigating common mechanisms involved in the pathogenesis of diabetic retinopathy and cognitive impairment in type 2 diabetes and determining whether retinal assessments could be useful to identify people with type 2 diabetes with MCI or at higher risk of developing cognitive decline or dementia. To fulfil these goals, prospective cross-sectional and longitudinal studies including people with type 2 diabetes aged 65 years or older were undertaken.

Herein, we present results of a cross-sectional analysis evaluating the relationship between MCI and retinal assessments of neurodysfunction and neurodegeneration. Our previous studies [31–33] suggested that retinal neurodysfunction, as determined by microperimetry, can discriminate people with diabetes with MCI from those with normocognition (NC). However, these results were preliminary, obtained using a limited sample size. Therefore, the main aim of the present study was to confirm or rule out these preliminary findings. In addition, other retinal parameters of neurodysfunction, such as those obtained by using a portable electroretinography device (RETeval), and measures of structural neurodegeneration, as determined by spectral domain optical coherence tomography (SD-OCT), were also evaluated.

## Methods

### Study design, study period and eligibility criteria

RECOGNISED (ClinicalTrials.gov registration no. NCT04281186) was undertaken by 11 clinical centres from seven European countries (Denmark, Italy, Montenegro, the UK, the Netherlands, Portugal and Spain). Ethical approval was obtained for the study (PR-AG272/2020), which was conducted according to the principles originating from the Declaration of Helsinki. Recruitment for the RECOGNISED cross-sectional study commenced on 16 November 2020 and was completed on 14 February 2022. Participants of the cross-sectional study who were enrolled also, subsequently, in the RECOGNISED longitudinal study were followed until 13 December 2024. Screening data obtained in both the cross-sectional and longitudinal studies were used for the analyses presented herein.

Eligibility criteria included: (1) people living with type 2 diabetes aged 65 years or older and able to provide written informed consent; (2) diabetes duration of at least 5 years; (3) no history of stroke or neurodegenerative disease; (4) early stages of diabetic retinopathy, from non-overt diabetic retinopathy (defined as no changes in the retina as observed by fundus examination and fundus photographs graded by masked graders at a reading centre) to moderate non-proliferative diabetic retinopathy (NPDR); (5) MoCA test <26 [34] and SAGE <17 [35].

People with: (1) previous history of stroke or neurodegenerative disease; (2) severe NPDR, proliferative diabetic retinopathy, diabetic macular oedema or any other eye disorder affecting the vision other than diabetic retinopathy; (3) previous laser photocoagulation; (4) a refractive error or −6 diopters or higher; (5) HbA_1c_ > 86 mmol/mol (10%); (6) a Geriatric Depression Scale-15 (GDS-15) score ≥9 (indicating major depression) [36]; (7) severe systemic diseases that would prevent the potential participant from completing the study; or (8) established dementia were excluded.

**Fig. 1:**
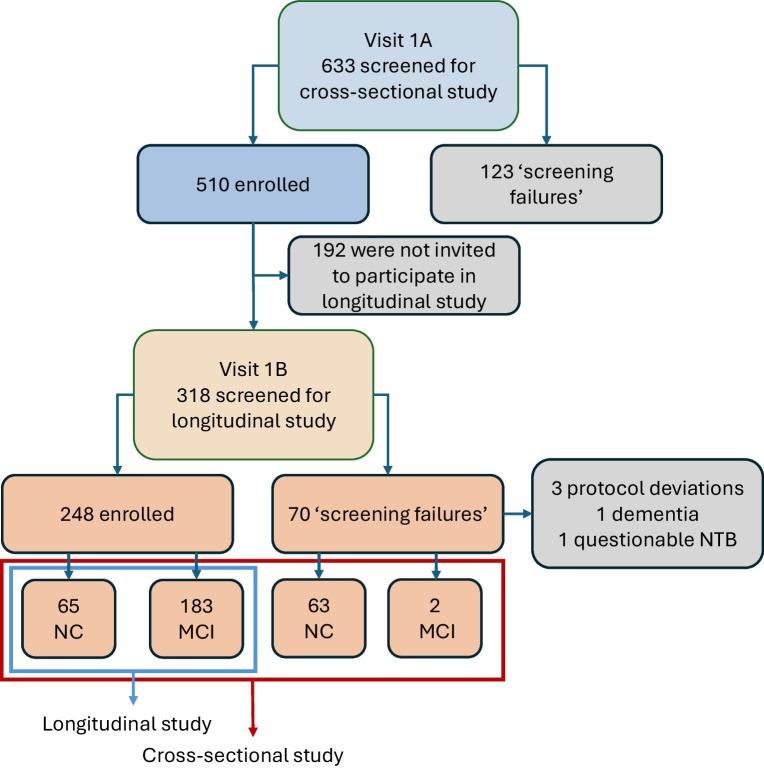
RECOGNISED flow chart showing the screening process with numbers of people included and excluded in the RECOGNISED cross-sectional and longitudinal studies. A total of 313 out of 633 screened individuals were included in the cross-sectional study. The 70 ‘screening failures’ accomplished the inclusion criteria and, in fact, were not included in the longitudinal study because they did not sign the informed consent to participate in the longitudinal study or the recruitment was already completed

Participants were identified in Endocrinology or Diabetes Units, following screening in Diabetic Eye Screening Programmes, or in Ophthalmic Clinics of all clinical sites involved in the study. Once a participant was screened, deemed eligible and enrolled in the RECOGNISED cross-sectional study, they were offered to take part in the RECOGNISED longitudinal study. Enrolment in the RECOGNISED longitudinal study followed a standard operating procedure, summarised in electronic supplementary material (ESM) Fig. 1, which also allowed the classification of participants in one of two groups: NC or MCI.

### Outcomes and assessments

Data obtained from the cross-sectional study and from the screening evaluation performed prior to participants being enrolled in the longitudinal study were used in the analyses presented herein (Fig. 1). A total of 128 participants with NC and 185 with MCI were included. The tests undertaken in the cross-sectional and longitudinal studies are presented in Table 1; for all, standard operating procedures were drawn up and followed. The main outcome assessed in this report was the ability of retinal parameters of neurodysfunction and/or neurodegeneration, with or without other characteristics, to identify people with MCI.

**Table 1:** All tests performed in the RECOGNISED cross-sectional and longitudinal studies

Variable	Prospective cross-sectional study	Prospective longitudinal cohort study					
Study phase		Baseline	Follow-up				
Visit number	1A	1B	2	3	4	5	6
Visit day/month	0 M	0 M	6 M	12 M	18 M	24 M	30 M
Allowed window	1–3 days	±1 M	±1 M	±1 M	±1 M	±1 M	±1 M
Procedures							
Informed consent for the clinical study	X	X					
Informed consent for biosamples	X						
History (medical and ocular)	X						
Inclusion/exclusion criteria	X	X					
Demographics	X						
Physical examination	X	X	X	X		X	X
Michigan Neuropathy Screening Instrument		X					X
Gait speed test (4 m)		X					X
BCVA	X		X	X		X	X
Microperimetry	X		X	X		X	X
Full-field flicker and photopic flash ERG and pupillary area (RETeval)		X		X			
Fundus photography^a^	X			X			
SD-OCT	X		X	X		X	X
OCTA^a^	X		X	X		X	X
Ultra-wide field fundus images^a^		X		X			X
Ultra-wide field FFA^a^		X		X			X
MoCA	X			X			X
SAGE	X			X			X
NTB		X		X			X
GDS-15	X			X			X
CDR		X		x			x
EQ-5D-3L questionnaire	X			X			X
NEI-VFQ-25	X			X			X
DSDRS	X						
MRI^b^		X					X
[18F]FDG PET^b^		X					X
Additional assessments							
Laboratory tests (blood and urine; fasting)	X			X		X	
Blood sampling (fasting), biobanking	X						
Non-drug therapy and concomitant medication	X	X	X	X	X	X	X
Adverse events	X	X	X	X	X	X	X
Telephone call					X		

^a^Graded, masked, by the staff at CORC

^b^Graded, masked, by the brain imaging reading centre

[18F]FDG PET, [18F]fluorodeoxyglucose positron emission tomography; FFA, fundus fluorescein angiography; M, month(s); OCTA, optical coherence tomography angiography

#### Cognitive assessment

At the first study visit (visit 1A; screening/baseline for the RECOGNISED cross-sectional study) the cognitive performance of participants was determined using screening tools: MoCA and SAGE. Measures of health-related and vision-related QoL were obtained (EuroQoL [EQ-5D-5L] [37] and the 25-item National Eye Institute Visual Function Questionnaire [NEI-VFQ-25] [38], respectively).

For the formal diagnosis of MCI or dementia, clinical criteria and additional neuropsychological assessments (beyond the MoCA and SAGE) are required [39]*.* For this purpose, in a second visit (visit 1B; screening/baseline for the RECOGNISED longitudinal study) a neuropsychological test battery (NTB) was performed to confirm that those individuals with MoCA and SAGE results suggestive of MCI (MoCA <26 and SAGE <17) indeed had MCI and were eligible to participate in the longitudinal study. The NTB covered the five main cognitive domains, including processing speed (Digit Symbol Substitution Test [DSST] and Trail Making Test [TMT]-A); attention and executive functioning (TMT-B, Wechsler Adult Intelligence Scale Digit Span [WAIS DS] backward, and letter fluency); memory (The Rey Auditory Verbal Learning Test [RAVLT]—direct, delayed, recognition, Rey–Osterreith Complex Figure Test [ROCF]—delayed, and WAIS DS forward); visuo-construction (ROCF-copy); and language (Boston Naming Test [BNT] and category fluency). Due to missing normative data for BNT in Italy and Portugal, the Battery for the Assessment of Severe Acquired Lexical Damage (BASALDI) in Italy, and the Paired Associates Learning (PAL-09) in Portugal were used instead. In addition, a Clinical Dementia Rating (CDR) [40] was performed.

Based on NTB and CDR testing, participants were classified as having NC or MCI. NC was defined by a normal NTB and CDR≤1. MCI was defined as impairment ≥1 of the five cognitive domains covered by the NTB and with a CDR≤1, both performed by a certified psychologist. A domain was defined as impaired if the average test performance was <15th percentile or when >50% of tests on the domain were <5th percentile based on local norm-references.

#### Diabetes Specific Dementia Risk Score

The Diabetes Specific Dementia Risk Score (DSDRS) was developed to help identify risk of dementia in people living with diabetes based on a set of predictors that are readily available in every patient’s medical record [41]. However, whether the DSDRS can discriminate people with diabetes with and without MCI is unknown. For this reason, we evaluated it in the present study.

#### Ophthalmological examinations

A total of 510 out of 633 participants screened for the cross-sectional study (visit 1A) were eligible and underwent fully refracted best corrected visual acuity (BCVA), microperimetry (Macular Integrity Assessment [MAIA] 3rd generation, CenterVue, Padova, Italy), SD-OCT (Cirrus OCT, Carl Zeiss, Oberkochen, Germany) and standardised seven field colour fundus photography (CFP) testing.

Subsequently, 313 individuals (128 with NC and 185 with MCI) who were eligible to participate in the longitudinal study underwent electrophysiology testing (RETeval, LKC Technologies, MD, USA) (visit 1B). In order to obtain comparable and reliable data and according to the International Conference on Harmonization Good Clinical Practice Standard Operating Procedure, a set of instructions was created to describe the procedures to perform electroretinography and assessment of the pupillary response using RETeval. Technicians and ophthalmologists undertaking this procedure were previously trained to ensure that this test was adequately and uniformly performed at all clinical sites participating in the study.

### Sample size

The sample size for the RECOGNISED cross-sectional and longitudinal studies was estimated, taking into account data from a previously conducted study [24]. Details of sample size calculations can be found in the ESM Methods.

### Masking

All endpoints from retinal images were obtained by masked graders at the Coimbra Ophthalmology Reading Centre (CORC), Portugal. Microperimetry and electroretinogram (ERG) data were obtained by endocrinologists/ophthalmologists/ophthalmic technicians (see also the Ophthalmological examinations section, above) without knowledge of the mental status of participants. Investigators obtaining other endpoints were not masked to participant group (MCI or NC). Participants were informed about their status and, thus, were unmasked also.

### Statistical analysis

For categorical variables, number and frequency were calculated; for quantitative variables, mean (±SD) and median (IQR) are given. Differences by cognitive status were estimated using χ^2^ test for qualitative variables and *t* test or Mann–Whitney *U* test for quantitative variables, depending on symmetry. Valid cases were considered when at least data regarding microperimetry and NTB were available (128 with NC and 165 with MCI). A sub-study in those participants in which RETeval was performed (81 with NC and 168 with MCI) was also undertaken.

Logistic regression models for MCI were performed. To find the best diagnostic combination, ophthalmological variables and participant characteristics such as age, gender, BMI, education level, HbA_1c_ and duration of diabetes were considered. A stepwise selection based on Akaike’s information criterion was carried out. ORs and 95% CIs are presented in tables. The performance of each model was assessed by plotting calibration curves. Net reclassification index (NRI) and the integrated discrimination improvement index (IDI) were also calculated. Youden index was used to determine the cut-off value for predicting the probability of MCI.

Since the visuo-construction domain was found to be the most commonly affected domain in people with diabetes with MCI, when compared with those with NC, we performed a post hoc analysis to examine whether there was a relationship between visuo-construction and parameters of retinal neurodysfunction and/or neurodegeneration independent of age, gender, diabetes duration and glycaemic control.

## Results

### General characteristics considering the cognitive status

Baseline characteristics of the people living with diabetes taking part in RECOGNISED, based in their cognitive status as assessed by NTB and CDR testing, are shown in Table 2. People with diabetes with MCI were statistically significantly older than those with NC (74.7±5.5 years vs 73.1±4.9 years; *p*=0.006) and had fewer years of education (10.3±4.7 vs 12.7±6.9; *p*<0.001); there were no statistically significant differences in diabetes duration, diabetes treatment and episodes of hypoglycaemia in the last 6 months.

**Table 2:** Clinical baseline characteristics of participants included in the current study considering cognitive status (NC vs MCI) assessed by NTB and CDR

Characteristic	NC *N*=128	MCI *N*=185	*p*
Age (years)	73.1±4.9	74.7±5.5	0.006
Gender (M/F)	65.2/34.8	65.2/34.8	0.99
Years of education^a^	12.7±6.9	10.3±4.7	<0.001
Diabetes duration (years)	19.5±10.3	18.7±8.8	0.68
Participants with hypoglycaemic episodes^b^ in the last 6 months (%)	18.0	18.4	0.92
Number of hypoglycaemic episodes^b^ in the last 6 months (%).	2.3±10.6	1.1±4.2	0.20
Fasting glucose, mmol/l	8.6±3.44	8.55±3.05	0.86
HbA_1c_, mmol/mol	56±1	57±1	0.57
HbA_1c_, %	7.3±0.9	7.4±1.1	0.57
HOMA-IR	3.63±2.61	3.70±3.21	0.84
Insulin treatment (%)	50.8	49.2	0.78
MoCA total score	24.8±2.9	21.1±3.9	<0.0001
DSDRS	5.60±2.2	6.85±2.6	<0.001
SAGE score	17.3±3.8	13.6±4.9	<0.0001
GDS-15 score	2.0±2.2	2.3±2.1	0.22
NEI-VFQ-25 total score	90.1±9.4	88.9±9.4	0.25
EQ-5D VAS (all items)	75.6±15.05	74.2±17.0	0.46
Performance for each cognitive domain			
Processing speed	51.01±19.94	35.50±20.90	<0.001
Attention and executive function	48.67±14.92	27.00±17.39	<0.001
Memory	40.90±18.04	25.80±15.12	<0.001
Visuo-construction	51.02±23.15	14.06±25.40	<0.001
Language	51.04±20.25	36.01±19.55	<0.001

Data are expressed as % or as mean ± SD

^a^Years of education: number of academic years a person completed in a formal programme provided by elementary and secondary schools, universities, colleges or other formal post-secondary institutions

^b^Capillary blood glucose < 3.89 mmol/l

F, female; M, male

Participants with MCI had lower MoCA and SAGE scores. The most common domain affected in the MCI group was visuo-construction (55.6% of individuals), followed by attention and executive function (33.3%), memory (32.1%), processing speed (26.7%) and language (23.5%). DSDRS was higher in participants with MCI than in participants with NC (6.8±2.6 vs 5.6±2.2; *p*<0.001). In the univariate logistic regression analyses, DSDRS was associated with the risk of MCI (OR 1.23; 95% CI 1.1, 1.35; *p*<0001). GDS-15, EQ-5D-3L and NEI-VFQ-25 scores were similar between groups.

### Relationship between retinal assessments of neurodysfunction, structural retinal neurodegeneration and cognitive status

The level of diabetic retinopathy in the Early Treatment Diabetic Retinopathy Study (ETDRS) classification was <35 in 84.1% and 81.1% of participants with NC and MCI, respectively (*p*=0.489) (Table 3).

**Table 3:** Results of the assessment of retinal neurodysfunction and neurodegeneration (right eye) in participants included in the current study considering cognitive status (NC vs MCI)

Variable	NC	MCI	*p*
Microperimetry and SD-OCT assessments	*N*=128	*N*=185	
MAIA-RS	24.8±2.8	23.5±4.3	0.01
MAIA-P1	85.4±21.3	79.1±23.2	0.001
MAIA-P2	94.1±13.5	91.1±14.0	<0.001
SD-OCT central retinal thickness (µm)	268.2±31.8	272.1±35.3	0.25
SD-OCT average macula thickness (µm)	278.5±17.7	279.0±17.0	0.90
SD-OCT macula volume (mm^3^)	10.0±0.6	10.0±0.6	0.99
SD-OCT average GCL–IPL thickness (µm)	77.0±7.6	74.9±11.5	0.07
SD-OCT minimum GCL–IPL thickness (µm)	71.7±12.7	69.1±16.3	0.20
SD-OCT average RNFL thickness (µm)	87.7±10.3	87.6±11.1	0.94
RETeval assessments	*N*=81	*N*=168	
Pupillary area ratio	1.61±0.4	1.76±0.4	0.002
Amplitude of flicker response (16 Tds stimulus) (µV)	16.3±7.6	15.2±7.8	0.25
Implicit time of flicker response (16 Tds stimulus) (ms)	30.9±3.7	31.6±4.4	0.28
Implicit time of b-wave (flash response) (ms)	30.0±2.5	30.3±4.7	0.052
Amplitude of b-wave (flash response) (µV)	23.6±11.0	20.7±12.2	0.03
Implicit time of a-wave (flash response) (ms)	12.62±2.26	13.0±3.82	0.97
Amplitude of a-wave (flash response) (µV)	−5.2 (−6.6; −3.0)	−4.6 (−6.8; −2.8)	0.29

Data are expressed as mean ± SD or median (IQR)

GCL–IPL, ganglion cell layer–inner plexiform layer; RNFL, retinal nerve fibre layer; Tds, stimulus flashes with constant retinal illuminance

Some parameters indicative of neurodysfunction were statistically significantly different between participants with MCI and those with NC (Table 3). Thus, participants with MCI had lower retinal sensitivity (MAIA for retinal sensitivity [MAIA-RS]) and gaze fixation (MAIA for fixation stability parameter P1 [MAIA-P1], MAIA for fixation stability parameter P2 [MAIA-P2], %) than participants with NC (23.5±4.3 vs 24.8±2.8; 79.1±23.2 vs 85.4±21.3; and 91.1±14.0 vs 94.1±13.5; *p*=0.01, *p*=0.001 and *p*<0.001, respectively). The pupillary area ratio (1.61±0.48 vs 1.76±0.44; *p*=0.002) as well as the photopic flash b-wave amplitude (23.6±11.0 µV vs 20.7±12.2 µV; *p*=0.03) were also statistically significantly different between people with diabetes with NC and those with MCI.

No statistically significant differences were observed between groups (MCI and NC) in any of the parameters of SD-OCT (Table 3).

### Multivariable analyses and prediction models

**Fig. 2:**
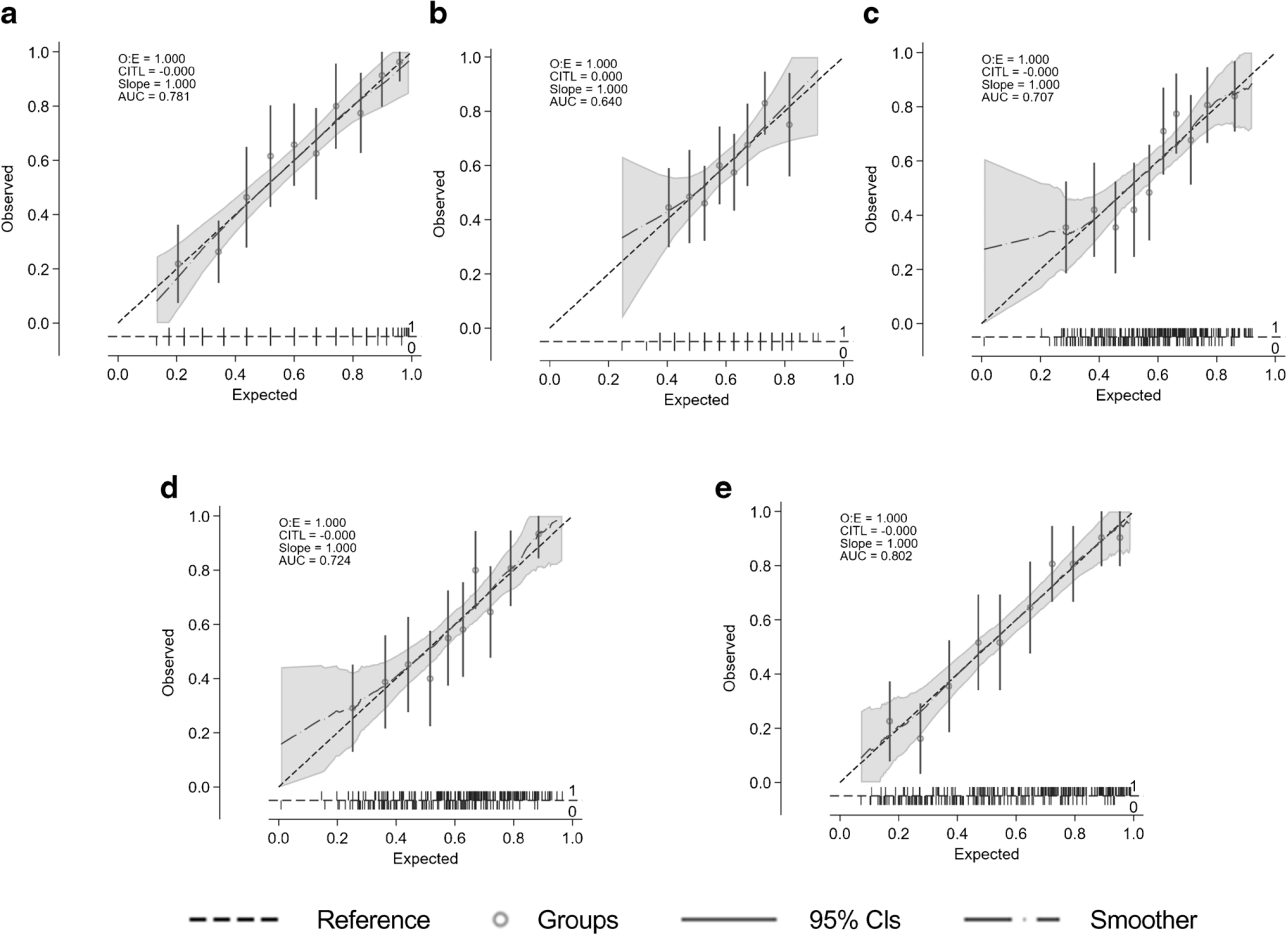
AUC values and calibration plots for predicting MCI in five models: (**a**) univariate MoCA; (**b**) univariate DSDRS; (**c**) multivariate model I, which includes MAIA parameters, years of education and DSDRS; (**d**) full multivariate model; (**e**) multivariate model I + MoCA. CITL, calibration in the large; O:E, observed:expected ratio

A multivariable logistic regression model was constructed with those clinical and ophthalmological variables presented in Tables 2 and 3 with a *p* value <0.1, with the exception of variables obtained with RETeval, as this test was undertaken only in those participants included in the longitudinal study (see Methods section). The variables independently associated with MCI were retinal sensitivity (MAIA-RS: OR 0.99; 95% CI 0.99, 1.00; *p*=0.065), fixation stability (MAIA-P1: OR 0.99; 95% CI 0.98, 1.00; *p*=0.058), years of education (OR 0.93; 95% CI 0.88, 0.98; *p*=0.004) and DSDRS (OR 1.18; 95% CI 1.07, 1.31; *p*=0.001). The AUC of this model was 0.71 (sensitivity 71.3%, specificity 66.6%). If MoCA score was added to the model, the AUC increased to 0.80 (sensitivity 64.64%, specificity 84.40%) (Table 4, Fig. 2). The models in which all variables were considered in the selection process (‘full model’) as well as NRI and IDI are displayed in ESM Tables 1 and 2.

**Table 4:** Multivariable logistic models fitted for those clinical and ophthalmological variables (right eye) of Table 2 and Table 3 with a *p* value <0.1

Variable	Model I for all of sample (AUC 0.71)			Model II for all of sample (AUC 0.80)		
	OR	(95% CI)	*p* value	OR	(95% CI)	*p* value
MAIA-RS RE (dB)×10	0.99	(0.99, 1.00)	0.065	1.002	(0.993, 1.011)	0.651
MAIA-P1 RE	0.99	(0.98, 1.00)	0.058	0.99	(0.98, 1.00)	0.106
Years of education	0.93	(0.88, 0.98)	0.004	0.98	(0.93, 1.03)	0.425
DSDRS	1.18	(1.07, 1.31)	0.001	1.16	(1.04, 1.29)	0.009
MoCA total score				0.74	(0.67, 0.81)	0.000

Variables considered but not independently related to MCI in the models: age, gender, BMI, HbA_1c_, diabetes duration and SD-OCT average ganglion cell layer–inner plexiform layer thickness

The cutpoint probability (sensitivity, specificity) for Model I was 0.57 (71.3, 66.6) and for Model II was 0.66 (0.64, 84.5)

RE, right eye

**Fig. 3:**
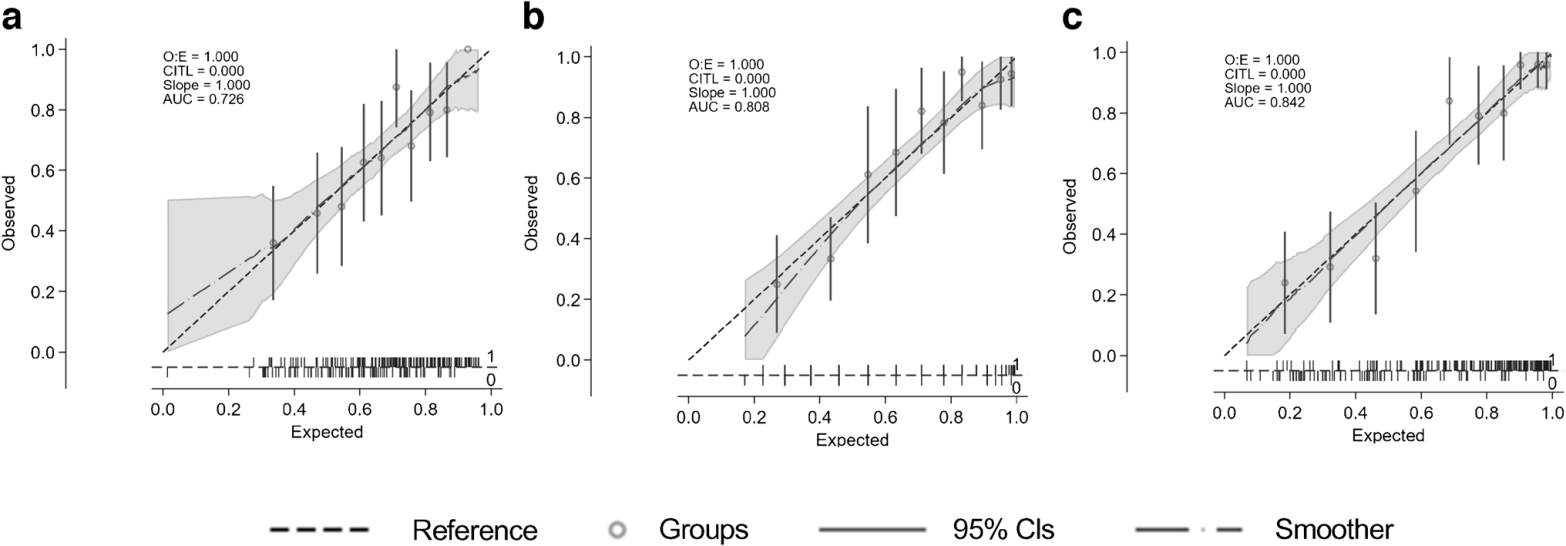
AUC values and calibration plots for predicting MCI in three models: (**a**) model I, which includes RETeval and MAIA parameters, years of education and DSDRS; (**b**) univariate model including MoCA; (**c**) model II, which includes the variables of model I plus MoCA. CITL, calibration in the large; O:E, observed:expected ratio

When the model was undertaken including only participants in whom RETeval was performed, the variables independently associated with MCI were pupillary area ratio (OR 2.57; 95% CI 1.31, 5.02; *p*=0.006), MAIA-RS (OR 0.99; 95% CI 0.98, 1.00; *p*=0.09), MAIA-P1 (OR 0.98; 95% CI 0.97, 1.00; *p*=0.02), years of education (OR 0.94; 95% CI 0.88, 1.00; *p*=0.04) and DSDRS (OR 1.17; 95% CI 1.04, 1.32; *p*=0.01). In this model, the AUC increased to 0.73 (sensitivity 68.3%, specificity 70.4%). When MoCA was added to the model, the AUC increased further to 0.84 (sensitivity 79.9, specificity 79.0), becoming the best model of all those tested (Table 5, Fig. 3).

**Table 5:** Multivariable logistic model including only participants in whom RETeval was performed

Variable	Model I for RETeval (AUC 0.72)			Model II for RETeval (AUC 0.84)		
	OR	(95% CI)	*p* value	OR	(95% CI)	*p* value
MAIA-RS RE (dB)×10	0.99	(0.98, 1.00)	0.092	1.002	(0.99, 1.01)	0.673
MAIA-P1 RE	0.98	(0.97, 1.00)	0.029	0.98	(0.97, 1.00)	0.029
Pupilar area ratio	2.57	(1.31, 5.02)	0.006	2.76	(1.34, 5.68)	<0.01
Years of education	0.94	(0.88, 1.00)	0.040	1.001	(0.94, 1.06)	0.970
DSDRS	1.17	(1.04, 1.32)	0.011	1.16	(1.04, 1.29)	0.012
MoCA total score				0.70	(0.63, 0.79)	<0.001

Variables considered but not independently related to MCI in the models: age, gender, BMI, HbA_1c_, diabetes duration, SD-OCT average ganglion cell layer–inner plexiform layer thickness, implicit time of b-wave and amplitude of b-wave (flash response)

The cutpoint probability (sensitivity, specificity) for Model I was 0.67 (68.3, 70.4) and for Model II was 0.62 (79.9, 79.0)

RE, right eye

**Fig. 4:**
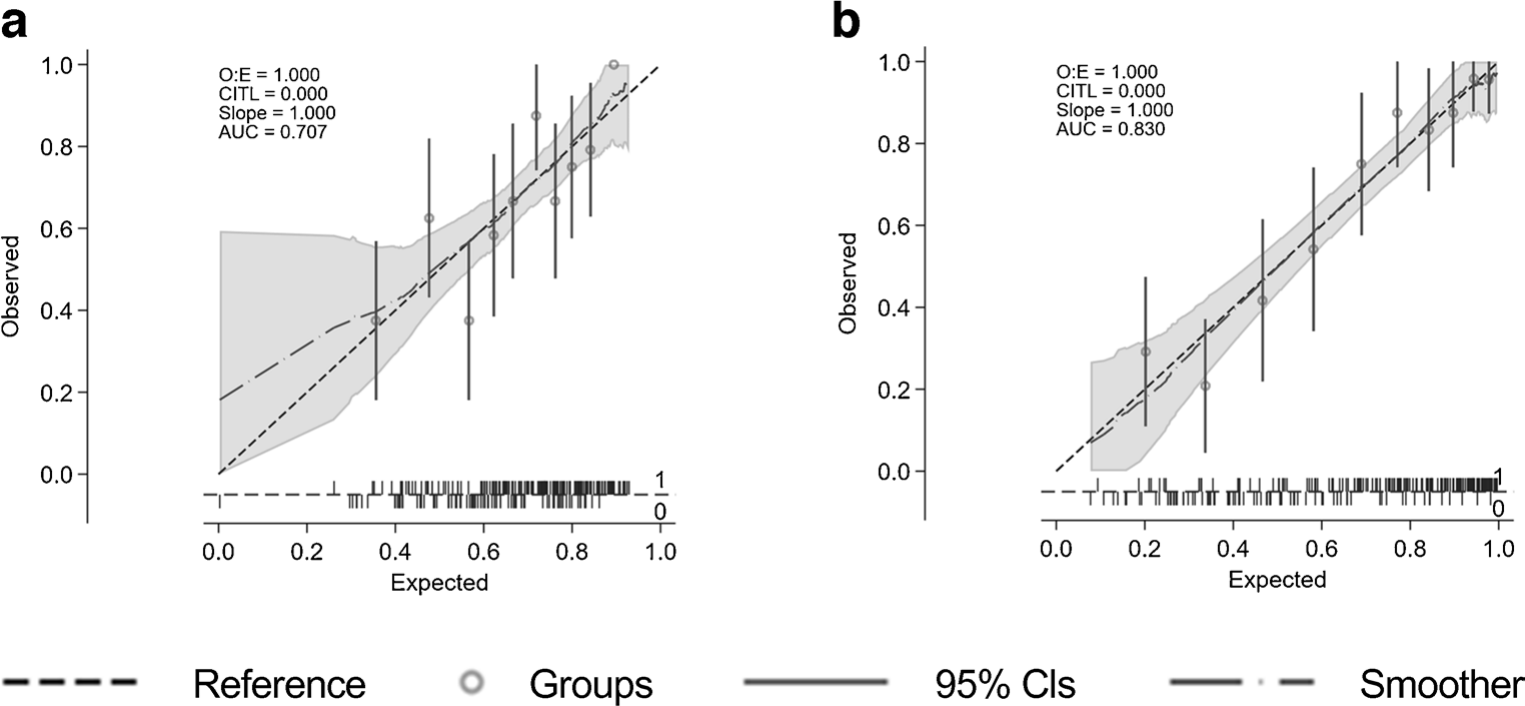
AUC values and calibration plots for predicting MCI in: (**a**) a model including RETeval parameters, years of education and DSDRS (model I), and (**b**) a model including the variables of model I plus MoCA. CITL, calibration in the large; O:E, observed:expected ratio

Given the potential feasibility and practicality, we also wanted to examine a prediction model based on RETeval, DSDRS and years of education (Table 6). The AUC of this model was 0.71 (sensitivity 60.0%, specificity 74.7%), increasing to 0.83 (sensitivity 76.3%, specificity 82.3%) when MoCA was included (Fig. 4).

**Table 6:** Prediction model based on RETeval, DSDRS and years of education

Variable	Model I for cases with RETeval done (AUC 0.71)			Model II for cases with RETeval done (AUC 0.83)		
	OR	(95% CI)	*p* value	OR	(95% CI)	*p* value
Pupilar area ratio RE	2.11	(1.12, 3.98)	0.021	2.64	(1.32, 5.26)	0.005
Amplitude b-wave RE (flash response) (µV)	0.98	(0.95, 1.00)	0.063	0.99	(0.96, 1.01)	0.304
DSDRS	1.15	(1.01, 1.30)	0.028	1.16	(1.01, 1.33)	0.037
Years of education	0.91	(0.86, 0.97)	0.004	0.99	(0.94, 1.06)	0.917
MoCA total score				0.72	(0.64, 0.80)	<0.001

Variables considered but not independently related to MCI in the models: age, gender, BMI, HbA_1c_, diabetes duration and implicit time of b-wave

The cutpoint probability (sensitivity, specificity) for Model I was 0.71 (60.0, 74.7) and for Model II was 0.67 (76.3, 82.3)

RE, right eye

### Relationship between retinal neurodysfunction, structural retinal neurodegeneration and visuo-constructive skills

The relationship between the most commonly affected endpoints, retinal neurodegeneration and visuo-construction, was compared considering age, gender, diabetes duration and glycaemic control. The main clinical features and retinal assessments in people with diabetes with or without impaired visuo-constructional skills are shown in Table 7.

**Table 7:** Baseline clinical variables and retinal neurodysfunction assessments (right eye) according to visuo-constructional skills in participants

Variable	Impaired visuo-constructional skills		*p* value
	No	Yes	
Total, *n* (%)	210 (67.1)	103 (32.9)	
Age (years)	73.8±5.2	74.8±5.8	0.10
Gender, *n* (%)			0.10
Female	68 (31.8)	43 (41)	
Male	146 (68.2)	62 (59)	
T2D duration (years)	18.9±9.9	19.2±8.5	0.51
HbA_1c_, mmol/mol	57±1	57±1	0.89
HbA_1c_, %	7.4±1.1	7.4±1.0	0.89
Fasting glycaemia, mmol/l	9.63±5.31	8.89±3.61	0.92
MAIA-RS (dB)	24.5±3.2	23.1±4.8	0.046
MAIA-P1 (%)	83.0±21.6	77.9±25.3	0.051
MAIA-P2 (%)	93.0±13.0	90.3±15.9	0.032
SD-OCT central retinal thickness (µm)	268.0±32.2	276.1±38.9	0.023
SD-OCT average macula thickness (µm)	277.4±17.0	281.3±18.0	0.11
SD-OCT macula volume (mm^3^)	10.0±0.6	10.1±0.6	0.11
SD-OCT average GCL–IPL thickness (µm)	75.6±8.9	75.5±12.8	0.68
SD-OCT minimum GCL–IPL thickness (µm)	70.1±14.1	69.3±18.1	0.61
SD-OCT average RNFL thickness (µm)	87.0±10.3	88.9±11.9	0.10
RETeval assessments	*N*=158	*N*=95	
Pupilar area ratio	1.7±0.5	1.7±0.4	0.38
Amplitude of flicker response (16 Tds stimulus) (µV)	16.2±7.9	14.7±7.7	0.11
Implicit time of flicker response (16 Tds stimulus) (ms)	31.1±4.6	32.0±3.4	0.38
Implicit time of b-wave (flash response) (ms)	30.1±3.6	30.4±5.0	0.64
Amplitude of b-wave (flash response) (µV)	22.5±11.7	19.9±12.2	0.047
Implicit time of a-wave (flash response) (ms)	12.6±2.7	13.4±4.4	0.22
Amplitude of a-wave (flash response) (µV)	−4.8 (−6.5; −2.8)	−4.5 (−6.8; −2.3)	0.36

Data are expressed as mean ± SD or median (IQR), unless otherwise stated

GCL–IPL, ganglion cell layer–inner plexiform layer; RNFL, retinal nerve fibre layer; T2D, type 2 diabetes; Tds, stimulus flashes with constant retinal illuminance

**Fig. 5:**
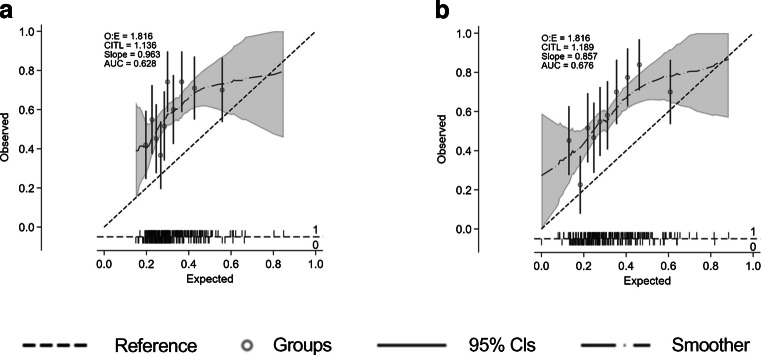
AUC values and calibration plots for predicting abnormal visuo-constructional skills in: (**a**) a model including MAIA and SD-OCT parameters (model I), and (**b**) a model including the variables of model I plus years of education. CITL, calibration in the large; O:E, observed:expected ratio

In a multivariable logistic regression analysis, the variables independently related to abnormal visuo-constructional skills were MAIA-RS (OR 0.91; 95% CI 0.86, 0.98; *p*=0.007), MAIA-P1 (OR 0.99; 95% CI 0.98, 1.00; *p*=0.06) and central retinal thickness (OR 1.01; 95% CI 1.00, 1.02; *p*=0.034). We did not find any independent relationship between the parameters assessed using RETeval and visuo-construction score. The AUC for this logistic regression model was 0.63 (sensitivity 73.5%, specificity 48.8%). When DSDRS and years of education were added to the model, the variables independently related to abnormal visuo-constructional skills were MAIA-RS (OR 0.94; 95% CI 0.88, 1.00; *p*=0.056), central retinal thickness (OR 1.01; 95% CI 1.00, 1.02; *p*=0.034) and years of education (OR 0.90; 95% CI 0.64, 0.80; *p*=0.0003) (Table 8). The AUC for this logistic regression model was 0.70 (sensitivity 70.4%, specificity 69.4%) (Table 8, Fig. 5).

**Table 8:** Multivariable logistic regression analysis showing the variables independently related to abnormal visuo-constructional skills

Variable	Model I for impaired visuo-constructional skills (AUC 0.63)			Model II for impaired visuo-constructional skills (AUC 0.70)		
	OR	(95% CI)	*p* value	OR	(95% CI)	*p* value
MAIA-RS RE (dB)	0.91	(0.86, 0.98)	0.007	0.94	(0.88, 1.00)	0.056
MAIA-P1 RE	0.99	(0.98, 1.00)	0.062			
SD-OCT central retinal subfield thickness RE	1.01	(1.00, 1.02)	0.027	1.01	(1.00, 1.02)	0.034
Years of education				0.90	(0.64, 0.80)	<0.001

Multivariable models have been fitted for those clinical and ophthalmological variables (right eye) displayed in Table 7 with a *p* value <0.1

Variables considered but not independently related to visuo-construction in the models: age, gender, BMI, HbA_1c_, diabetes duration and amplitude of b-wave (flash response)

The cutpoint probability (sensitivity, specificity) for Model I was 0.28 (73.5, 48.8) and for Model II was 0.32 (70.4, 69.4)

RE, right eye

Finally, we observed that participants with abnormal visuo-constructional skills had worse QoL scores, as determined by the EQ-5D-5L visual analogue scale (VAS), than those with a normal score in this domain (71.1±17.3 vs 76.5±15.4; *p*=0.005).

## Discussion

Extending our previous observations [31–33], we confirmed in this prospective, European, multicentre RECOGNISED cross-sectional study that retinal sensitivity and gaze fixation, measured by microperimetry, are useful parameters to identify MCI in people living with diabetes. We also provided novel evidence on the usefulness of photopic ERG and assessment of pupillary area, both determined by a portable hand-held diagnostic device (RETeval), to identify MCI. These findings are important because the current diagnosis of cognitive impairment is based on neuropsychological tests that are complex and time-consuming, making their incorporation into clinical practice unfeasible. In contrast, the aforementioned retinal assessments can be performed in a relatively short period of time, around 10 min per eye for microperimetry and less than 30 s per eye for RETeval. Additionally, the validity of RETeval is independent of depression and mood, thus addressing these potentially confounding variables in people living with diabetes.

Since there are still no clear therapeutic strategies for preventing or arresting the progression of MCI to dementia, the question of the benefit and potential impact of early identification of MCI in people with diabetes may arise. By uncovering cognitive dysfunction, appropriate support can be provided to patients, which would improve treatment adherence and diabetes self-management, resulting subsequently in better glycaemic control, less severe hypoglycaemic episodes, reduction in number of hospital admissions and decreased overall morbidity and mortality [15, 16]. For these reasons, the diagnosis of cognitive impairment is not only recommended, but will enable a more personalised management for people living with type 2 diabetes. Indeed, the ADA recommends individualising diabetes treatment, taking into account the cognitive capacity of patients [18].

Among the functional retinal endpoints studied addressing neurodysfunction, we found retinal sensitivity (MAIA-RS) and gaze fixation (MAIA-P1), as measured by microperimetry, and pupillary area ratio and photopic flash b-wave amplitude, as determined by RETeval, to be statistically significantly different between people with diabetes with MCI and those with NC. In contrast, no statistically significant differences were found in the parameters assessed by SD-OCT, suggesting that there was no difference in structural neurodegeneration of the retina between the two groups. This is not surprising because it would be expected that neurodysfunction should precede retinal neurodegeneration, in which thinning of the neuroretina would occur as a result of neuronal loss, as has been previously reported in early stages of diabetic retinopathy [24–26]. Since a non-diabetic control group has not been included in the study we cannot confirm this already well-established observation. Regarding the b-wave, it should be mentioned that it is the positive deflection following the a-wave, which originates from the depolarisation of inner retinal Müller and bipolar cells, suggesting a functional alteration in people with diabetes with MCI. This deficit is likely due, at least partly, to a generalised reduction in excitatory neurotransmission of retinal ON bipolar cells [42]. Since glial activation or reactive gliosis is a common feature in the brain and in the retina in diabetes-induced cognitive impairment [43], it could be speculated that this could contribute also to the reduction of b-wave amplitude in individuals with MCI.

Another interesting finding was the statistically significant difference in DSDRS observed between participants with NC and those with MCI. DSRDS was proposed in 2013 by Exalto et al [41] as a risk score for the 10 year prediction of dementia in individuals with type 2 diabetes, but some reports suggest that it could be useful for identifying people with diabetes with MCI [44, 45]. The results of the current study confirm this. Since the variables needed to calculate DSRDS (i.e. age, gender, education, history of diabetic foot, acute metabolic events, depression, microvascular disease, cardiovascular disease and cerebrovascular disease) are relatively easy to obtain from medical records, the incorporation of this score into the current clinical practice to identify people with diabetes with MCI should be feasible and immediately implementable in clinical practice.

Overall, multivariable analyses and prediction models showed that the above neuroretinal assessments combined with simple clinical variables, such as years of education and those included in DSRDS, resulted in acceptable levels of MCI predictability (AUC 0.72, sensitivity 68.3, specificity 70.4), increasing to very acceptable levels when the MoCA was included into the model (AUC 0.84, sensitivity 79.9, specificity 79.0). This strategy could significantly optimise the use of current time-consuming NTBs, which require specialised psychologists, making them very difficult, if not impossible, to implement in clinical practice. However, it could be argued that microperimetry and RETeval are not available in the vast majority of centres and that an accurate cost-effectiveness analysis would be needed before these strategies are introduced. In this regard, it should be noted that the microperimetry equipment is more expensive and requires some training of the healthcare professionals using it and of the patients receiving it, and the test itself takes longer than RETeval testing. In addition, the portability and small size of RETeval are notable advantages when comparing it with microperimetry. For this reason, we calculated the predictability of MCI in people with diabetes by including in the model only parameters independently related to MCI obtained with RETeval (i.e. pupillary area ratio and photopic flash b-wave amplitude), besides years of education and DSRDS. The AUC was 0.71 (sensitivity 60.0%, specificity 74.7%), which increased to 0.83 (sensitivity 76.3%, specificity 82.3%) when MoCA was included in the model. These results open up a new feasible strategy screen for MCI in people living with type 2 diabetes.

As previously reported in the same cohort of participants, we found that visuo-construction was the most affected cognitive domain [46]. There is some evidence that rapid cognitive decline may be better predicted by impairment in the visuo-construction domain in the general population [47–49], but little was known as to whether this was the case in people living with diabetes [50]. Visuo-constructional impairment refers to difficulties in perceiving and reproducing spatial relationships and constructing visual–spatial representations. This impairment primarily affects the parietal and occipital lobes of the brain, which are responsible for visual processing and spatial awareness. As a result, the ability to accurately copy or draw objects, recognise shapes and patterns and complete visual puzzles is compromised. This can interfere with recreational activities, limit participation in hobbies and strain interpersonal relationships. These difficulties can lead to frustration and reduced independence and QoL. Indeed, we found that people with diabetes with abnormal visuo-constructional skills presented with worse QoL, as determined by the EQ-5D-5L VAS. This result agrees with previous studies performed in the general population, which suggested a critical role of impaired visuospatial abilities in the genesis of a deficit in carrying out daily living activities [51, 52]. In addition, in the present study, we provide the first evidence of an independent relationship between visuo-construction test skills, using the ROCF test, and retinal parameters (retinal sensitivity and gaze fixation). This finding is not surprising given that microperimetry examines not only the functional status of the retina but also of the entire visual system, and domains involved in short-term memory, adequate perceptual speed and executive function are also required for its execution. Interestingly, these domains are also needed to perform the ROFC test. Furthermore, it is worth mentioning that type 2 diabetes affects all these domains and, therefore, microperimetry could be useful for testing all of them in an integrative manner.

Notably, years of education remained independently related to MCI and abnormal visuo-constructional skills besides retinal assessments indicative of retinal neurodysfunction, MoCA and DSDRS. This is aligned with the results of a recent meta-analysis showing that education has a delaying effect against cognitive decline progression, and in particular from risk of progression from subjective cognitive decline to MCI [53]. In fact, education is a major contributor to the enhancement of cognitive reserve, which enables individuals to better counteract the gradual brain changes associated with ageing and neurodegenerative diseases, as well as their related symptoms [54].

Strengths of this study include the prospective study design, the inclusion of people living with diabetes recruited from many sites across Europe, the standardisation of all diagnostic technologies used, following study-specific SOPs, and the unbiased collection and grading of endpoints. All neuropsychological tests were done by expert neuropsychologists. Both retinal neurodysfunction and neurodegeneration were combined with a complete NTB which, to the best of our knowledge, had not been previously undertaken for the identification of people with diabetes with MCI. Potential limitations include its cross-sectional design (results of the RECOGNISED longitudinal study will be presented in the future) which does not allow causal associations to be examined. Only people with diabetes with no overt or just early stages of diabetic retinopathy were included, thus preventing a general extrapolation. Nevertheless, it should be noted that the vast majority of people living with diabetes aged over 65 years present within the diabetic retinopathy limits of the current study. Finally, apart from years of education, social determinants were not measured and, therefore, their potential influence in cognitive measures cannot be ruled out.

In conclusion, retinal neurodysfunction is related to MCI and the loss of visuo-construction abilities. These results support the use of microperimetry and/or RETeval in combination with simple clinical variables as a new strategy to identify people with diabetes with MCI. Since the parameters obtained with these tools are less influenced by mood and depression, it seems that this approach could optimise current screening of MCI in people living with diabetes.

## Supplementary Information

Below is the link to the electronic supplementary material.

Supplementary file1 (PDF 220 KB)

## Data Availability

The datasets generated during and/or analysed in the current study are available from the corresponding authors upon reasonable request.

## Abbreviations

BCVA: Best corrected visual acuity
CDR: Clinical Dementia Rating
CORC: Coimbra Ophthalmology Reading Centre
DSDRS: Diabetes Specific Dementia Risk Score
DSST: Digit Symbol Substitution Test
ERG: Electroretinogram
GDS-15: Geriatric Depression Scale-15
IDI: Integrated discrimination improvement index
MAIA: Macular Integrity Assessment
MAIA-P1: Macular Integrity Assessment for fixation stability parameter P1
MAIA-P2: Macular Integrity Assessment for fixation stability parameter P2
MAIA-RS: Macular Integrity Assessment for retinal sensitivity
MCI: Mild cognitive impairment
MMSE: Mini Mental State Examination
MoCA: Montreal Cognitive Assessment
NC: Normocognition
NEI-VFQ-25: 25-item National Eye Institute Visual Function Questionnaire
NPDR: Non-proliferative diabetic retinopathy
NRI: Net reclassification index
NTB: Neuropsychological test battery
QoL: Quality of life
RAVLT: Rey Auditory Verbal Learning Test
ROCF: Rey–Osterrieth Complex Figure
SAGE: Self-Administered Gerocognitive Examination
SD-OCT: Spectral domain optical coherence tomography
TMT: Trail Making Test
VAS: Visual analogue scale
