# Supplementary material for: Relationship between retinal neurodysfunction and cognitive impairment in type 2 diabetes: results of the RECOGNISED cross-sectional study
Source: Diabetologia. 2026 Jan 29;69(5):1337–53. doi: 10.1007/s00125-025-06664-4 (PMC13005843; doi:10.1007/s00125-025-06664-4)
Supplement: Supplementary file 1 — Supplementary file1 (PDF 220 KB) [file 125_2025_6664_MOESM1_ESM.pdf]

ELECTRONIC SUPPLEMENTARY MATERIAL

Title: Relationship between retinal neurodysfunction and cognitive impairment in type 2 diabetes: Results of the RECOGNISED cross-sectional study.

Simó R, et al.

ESM Figure 1. Standard Operating Procedure (SOP)

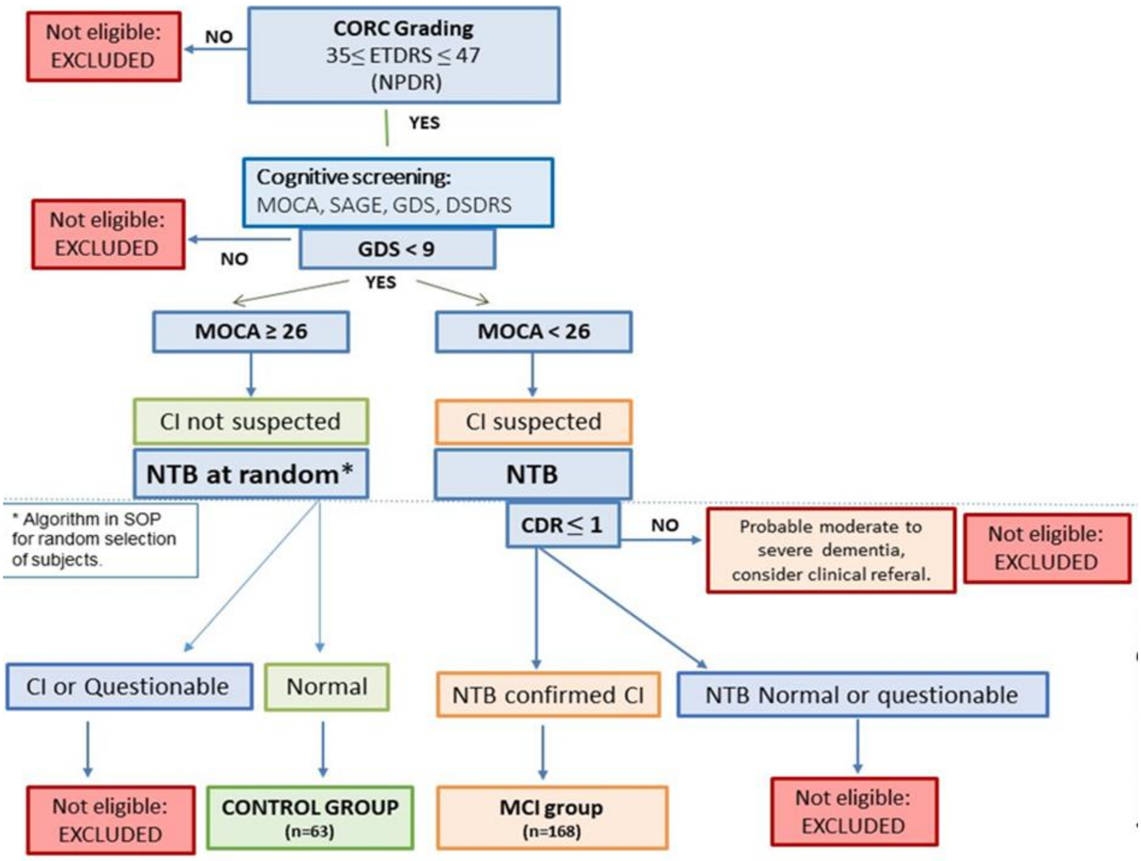

ESM Methods

Sample size justification

The sample size (n) was calculated using the following formula: n = (Zα/2+Zβ)2 *2*σ2 / d2, where Zα/2 is the critical value of the normal distribution at α/2 (for a confidence level of 95%, α is 0.05 and the critical value is 1.96), Zβ is the critical value of the normal distribution at β (for a power

of 80%, β is 0.2 and the critical value is 0.84), σ2 is the population variance, and d is the difference we would like to detect.

Taking into account the results obtained in a previous study of the consortium based on scotopic microperimetry [1], a difference in retinal sensitivity of 2 dB as being clinically relevant, and the variance obtained in PLWD with MCI being 16. With these considerations, the minimum sample size required to detect a difference in retinal sensitivity between PLWD with MCI and with rapid cognitive impairment and/or dementia conversion vs. non-progressors/slow progressors subjects is 63 for each group. The expected percentage of MCI-T2D patients that will convert to dementia during the follow-up (30 months) will be around 50%. Thus, the number of MCI-T2D patients included at baseline was estimated to be 126.

Taking into account a 25% drop-out rate, 168 PLWD with MCI were included in the prospective study. In addition, a group of normo-cognitive PLWD (n=63) was also included in order to evaluate the eventual decline in retinal and cognitive parameters over time during the course of the follow-up (this group will act as a control group).

Based on a previous study [2] in which the rate of MCI in PLWD was around 20% and taken into account that the prevalence of NPDR is at least of 30% in this population, we would need to screen medical records of ~2400 subjects in order to identify the potentially eligible ~700 participants for the cross-sectional study that are required for the identification of the 168 eligible subjects with MCI required for the prospective longitudinal cohort study.

1. Ciudin A, Simó-Servat O, Hernández C, et al. (2017) Retinal Microperimetry: A New Tool for Identifying Patients With Type 2 Diabetes at Risk for Developing Alzheimer Disease. Diabetes, 66(12): 3098–104. DOI: 10.2337/db17-0382

2. Koekkoek PS, Janssen J, Kooistra M, et al. (2016) Case-finding for cognitive impairment among people with Type 2 diabetes in primary care using the Test Your Memory and Self-Administered Gerocognitive Examination questionnaires: the Cog-ID study. Diabetic Medicine, 33(6): 812–9. DOI: 10.1111/dme.12874

ESM Table 1. Full model

VARIABLE		OR	(95%CI)	p-value
Age (years)		0.93	(0.86; 1.01)	0.0825
Sex	Female	1		0.7116
	Male	1.11	(0.64; 1.92)	
Years of Education		0.93	(0.88; 0.98)	0.0105
DSDRS_score		1.34	(1.12; 1.59)	0.0010
MAIA RS		0.99	(0.98; 1.00)	0.0633
MAIA P1		0.97	(0.94; 1.00)	0.0658
MAIA P2		1.03	(0.98; 1.09)	0.2355
SD-OCT Macular Thickness (µm)_		1.01	(0.99; 1.02)	0.3718
SD-OCT Macular Thickness Avg Cube (µm)		0.90	(0.75; 1.08)	0.2775
SD-OCT Average GCL+IPL Thickness_(µm)		0.98	(0.92; 1.04)	0.5194
SD-OCT Minimum GCL+IPL Thickness(µm)		1.01	(0.97; 1.05)	0.6511
SD-OCT Average RNFL Thickness_(µm)		1.01	(0.98; 1.04)	0.4250

ESM Table 2. Net reclassification index (NRI) and Integrated Discrimination Improvement (IDI) for the adjusted models

Model 1	Model 2	NRI	lim inf NRI	Lim sup NRI	IDI	lim inf IDI	Lim sup IDI
DSDRS_score,	Years of education MAIAP1_ MAIARS	0,167	0,162	0,172	0,060	0,034	0,085
DSDRS_score yearsofeducation MAIAP1 MAIARS	age gender MAIAP2 macular_thick macular_thick_aver_cube macula_vol_cube aver_GCLIPL_thick min_GCLIPL_thick aver_RFNL_thick	0,008	-1,588	1,603	0,022	0,006	0,037

thick: thickness, aver: average; min: minimum
